# Examination of PHB Depolymerases in *Ralstonia eutropha*: Further Elucidation of the Roles of Enzymes in PHB Homeostasis

**DOI:** 10.1186/2191-0855-2-26

**Published:** 2012-04-26

**Authors:** Christopher J Brigham, Esther N Reimer, ChoKyun Rha, Anthony J Sinskey

**Affiliations:** 1 Department of Biology, Massachusetts Institute of Technology, Cambridge, MA, 02139, USA; 2 RWTH Aachen University, 52062, Aachen, Germany; 3 Biomaterials Science and Engineering Laboratory, Massachusetts Institute of Technology, Cambridge, MA, 02139, USA; 4 Division of Health Sciences Technology, Massachusetts Institute of Technology, Cambridge, MA, 02139, USA; 5 Engineering Systems Division, Massachusetts Institute of Technology, Cambridge, MA, 02139, USA

**Keywords:** *Ralstonia eutropha*, Polyhydroxyalkanoates, Polyhydroxybutyrate, Biomaterials, Depolymerase, Granules, Carbon utilization, Electron microscopy, Stereology

## Abstract

Polyhydroxyalkanoates (PHA) are biodegradable polymers that are attractive materials for use in tissue engineering and medical device manufacturing. *Ralstonia eutropha* is regarded as the model organism for PHA biosynthesis. We examined the effects of PHA depolymerase (PhaZ) expression on PHA homeostasis in *R. eutropha* strains. In order to analyze the impact of PhaZs on *R. eutropha* granule architecture, we performed electron microscopy on several *phaZ* knockout strains and the wild type strain grown under PHA production conditions. Analysis of the acquired micrographs was based on stereology: the ratio of granule area and cell area was determined, along with total granule count per full-size cell image. Cells bearing a *phaZ2* knockout mutation alone or in conjunction with a *phaZ1* mutation were found to have a high granule volume per cell volume and a higher granule count compared to wild type. A *phaZ* quadruple knockout strain appeared to have a low granule volume per cell volume and a low granule count per cell. Cells bearing a *phaZ3* knockout were found to have a higher granule count than the wild type, whereas granule volume per cell volume was similar. Accordingly, we hypothesize that PhaZs have not only an impact on PHA degradation but also on the 3-dimensional granule architecture. Based on our data, PhaZ2 is postulated to affect granule density. This work increased our knowledge about PHA depolymerases in *R. eutropha*, including enzymes that had previously been uncharacterized.

## Introduction

Polyhydroxyalkanoates (PHA) are carbon storage molecules synthesized by many microorganisms during times of (non-carbon) nutrient stress. One of the most well-studied members of the PHA family is the homopolymer polyhydroxybutyrate (PHB). Several species of bacteria have been shown to produce PHB (see ([Anderson and Dawes 1990]; [Rehm 2003]; [Stubbe et al. 2005]; [Sudesh et al. 2000]) for reviews), including *Ralstonia eutropha,* which is considered the model organism for study of PHA biosynthesis ([Reinecke and Steinbüchel 2009]). *R. eutropha* grows autotrophically ([Ishizaki et al*.* 2001]; [Volova and Voinov 2003]) or heterotrophically on carbon substrates such as fructose ([Tian et al. 2005b]), organic acids ([Yang et al. 2010]), or fatty acids/lipids ([Brigham et al. 2010]) and has been demonstrated to produce PHB under nitrogen or phosphate limitation. PHA, including but not limited to PHB, have been shown to exhibit a range of thermal and physical properties similar to petroleum-based plastics ([Budde et al*.* 2011]; [Sudesh et al. 2000]), and have also been shown to be biodegradable ([Shishatskaya et al*.* 2005]).

Both the ubiquity of PHB, and various PHA types, and the production of low molecular weight PHB in a range of organisms, including mammals ([Reusch 1989]), have led researchers to extensive examination of PHAs for use in medical materials and devices. PHAs have been demonstrated to be biocompatible and have been used in surgical procedures ([Shishatskaya et al*.* 2002a]; [Shishatskaya et al*.* 2002b]; [Shishatskaya et al*.* 2004]; [Sodian et al. 1999]). The immunological and histological responses of tissues and organisms towards PHA implants have demonstrated favorable results ([Sevastianov et al*.* 2003]; [Shishatskaya et al*.* 2004]; [Volova et al. 2003]). PHA is currently being used to produce commercially available sutures and meshes that have shown to exhibit many promising characteristics, including durability, tensile strength, flexibility, and resorbability (http://www.tepha.com). PHA matrices are also being examined for encapsulation of bioactive agents, such as tumor drugs and antibiotics, to be inserted and delivered at the site of tissue distress ([Chee et al*.* 2008]). Many PHA types have been used as growth substrata for cells in tissue engineering ([Chen and Wu 2005]). In earlier studies, a copolymer of polyglycolic acid (PGA) and PHB was used to produce pulmonary valve leaflets and pulmonary artery scaffolds in sheep ([Shum-Tim et al. 1999]). This study was followed up by construction of a PHA-based heart valve scaffold, which was again surgically inserted into sheep ([Sodian et al. 1999]). Both of these early studies illustrated that tissue engineering using biopolymer scaffolds is possible. Fibroblast cells from the NIH 3T3 line have been shown to exhibit growth on PHB and PHA copolymer substrates. These polymers have good functional properties in both an *in vitro* and *in vivo* setting ([Shishatskaya et al*.* 2008]). PHB composites have been constructed, such as PHB with hydroxyapatite for bone tissue engineering ([Chen and Wu 2005]). PHA, specifically a copolymer of 3-hydroxybutyrate and 4-hydroxybutyrate (P(3HB-co-4HB)), shown to be a promising substrate for growing rabbit smooth muscle cells and demonstrated high elastin formation, suggesting efficacy of PHA in tissue engineering ([Cheng et al*.* 2008]).

Even with the number of important discoveries that have been made regarding *R. eutropha* PHB metabolism, physiology, and biochemistry over the past few decades, there are still attributes of PHB metabolism and intracellular granule formation that remain to be fully explained. In this work, we examine PHB depolymerase enzymes, termed PhaZ, and their role in *R. eutropha* PHB utilization and intracellular PHB granule formation. Table 1 lists established and putative PHA depolymerases in *R. eutropha.* The principle depolymerase enzyme of *R. eutropha*, PhaZ1, has been well-studied and plays a significant role in PHB mobilization ([Uchino and Saito 2006]; [Uchino et al. 2007]; [York et al. 2003]). Another depolymerase enzyme, PhaZ2, has been shown to degrade PHB intracellularly ([York et al. 2003]). It is important to note that, in published research prior to publication of the *R. eutropha* H16 genome sequence in 2006, what is now catalogued as PhaZ5 was referred to as PhaZ3. A new and currently uncharacterized PhaZ3 enzyme is now referenced. Two depolymerase candidates, PhaZ3 and PhaZ5, exhibit homology to other intracellular depolymerases and are even classified in the PHA Depolymerase Engineering Database (http://www.ded.uni-stuttgart.de/) as intracellular, short-chain PHA depolymerases that act on native PHA ([Knoll et al*.* 2009]). One candidate, PhaZ5 (formerly PhaZ3) has been discussed in previous works ( [York et al.2003]), while PhaZ3 has not been characterized to date. Here, we describe the PHB mobilization phenotype of a *phaZ3* mutant strain, alone or in conjunction with other *phaZ* gene deletions. We also examine expression of *phaZ* genes and determine if absence or overexpression of specific *phaZ* genes influences PHB homeostasis. Lastly, we examine *phaZ* mutant strains by electron microscopy and suggest PHB remodeling for PhaZ enzymes associated with PHB granules.

**Table 1:** ***R. eutropha*PHA depolymerase and related enzymes**

**Name**	**Function**	**PHA Depolymerase Superfamily designation**^**a**^	**Protein ID**^**b**^	**Locus tag**
PhaZ1	Intracellular PHA depolymerase	i-nPHAscl (no lipase box)	CAJ92291.1	H16_A1150
PhaZ2	Intracellular PHA depolymerase	i-nPHAscl (no lipase box)	CAJ93939.1	H16_A2862
PhaZ3	Putative intracellular PHA depolymerase	i-nPHAscl (no lipase box)	CAJ95139.1	H16_B0339
PhaZ4	Putative PHA depolymerase	i-nPHAscl (no lipase box)	AAP85930.1	PHG178
PhaZ5	Intracellular PHA depolymerase	i-nPHAscl (no lipase box)	CAJ95805.1	H16_B1014
PhaZ6^c^	PHA depolymerase	e-dPHAscl (catalytic domain type 1)	CAJ96855.1	H16_B2073
PhaZ7	PHA depolymerase	e-dPHAscl (catalytic domain type 1)	CAJ97183.1	H16_B2401
PhaY1	*D*-(−)-3-hydroxybutyrate oligomer hydrolase	N/A^d^	CAJ93348.1	H16_A2251
PhaY2	*D*-(−)-3-hydroxybutyrate oligomer hydrolase	N/A^d^	CAJ92475.1	H16_A1335

^a^PHA Depolymerase Superfamily designation according to the PHA Depolymerase Engineering Database (http://www.ded.uni-stuttgart.de/). Putative primary protein sequences of each PHA depolymerase were used as seed sequences for a BLAST search of enzymes present in the database. Each depolymerase candidate is also classified according to a superfamily, as discussed in ([Knoll et al. 2009]).

^b^From DNA Data Bank of Japan (http://www.ddbj.nig.ac.jp/).

^c^Originally characterized as PhaZd ([Abe et al*.* 2005]).

^d^Sequence characteristics not present in PHA Depolymerase Engineering Database.

## Materials and methods

### Bacterial strains, plasmids and primers

The bacterial strains used in this work are listed in Table 2. Oligonucleotide primers used in this study are listed in Table 2. All plasmids for overexpression of *phaZ* genes were derived from the broad host range vector pBBR1MCS-2 (Table 2). The *Escherichia coli* strain TOP10 was used for the amplification of the plasmids throughout the cloning process, and *E.coli* strain S17-1 was used for conjugal transfer of plasmids into *R. eutropha* H16.

**Table 2:** Bacterial strains and plasmids used in this study

**Strain or Plasmid**	**Description**	**Reference**
***R. eutropha*****strains**		
H16	Wild type, Gm resistant	ATCC 17699
Re1097	H16/Δ*phaZ1*	([York et al. 2003])
Re1107	H16/Δ*phaZ5*	([York et al. 2003])
Re1110	H16/Δ*phaZ2*	([York et al. 2003])
Re1111	H16/Δ*phaZ1*Δ*phaZ*Δ*phaZ5*	([York et al. 2003])
Re1112	H16/Δ*phaZ1*Δ*phaZ2*	([York et al. 2003])
Re2005	H16/Δ*phaZ1*Δ*phaZ2*Δ*phaZ3*Δ*phaZ5*	This study
Re2006	H16/Δ*phaZ3*	This study
*E. coli*strains		
Top10	IF^-^*mcrA* Δ(*mrr-hsdRMS-mcrBC*) φ80*lacZ*ΔM15 Δ*lacX*74 *recA*1 *araD*139 Δ(ara-leu) 7697 *galU galK rpsL* (Str^R^) *endA*1 *nupG* λ-	Invitrogen
S17-1	*recA pro hsdR* RP4-2-Tc::Mu-Km::Tn7	ATCC47055; ([Simon et al. 1983])
Plasmids		
pBBR1MCS-2	Broad host range cloning vector, confers Km resistance	([Kovach et al. 1995])
pER1	pBBR1MCS-2 with *phaZ1* inserted into Kpn1/Xba1 restriction sites, confers Km resistance	This study
pER2	pBBR1MCS-2 with *phaZ2* inserted into Kpn1/Xba1 restriction sites, confers Km resistance	This study
pER3	pBBR1MCS-2 with *phaZ3* inserted into Sal1/Xba1 restriction sites, confers Km resistance	This study
pER4	pBBR1MCS-2 with *phaZ5* inserted into Kpn1/Xba1 restriction sites, confers Km resistance	This study
pGY46	*phaC* precise-deletion gene replacement plasmid, confers Km resistance	([York et al. 2001])
pCJB3	pGY46 with regions upstream and downstram of phaZ3 inserted between SacI and XbaI	This study

### Materials

All chemicals were purchased from Sigma-Aldrich, (St. Louis, MO, USA) unless indicated differently below. The following kits were used according to the instructions given by the manufacturer: The QIAquick® Gel Purification Kit (Qiagen, Valencia, CA) for gel purification of DNA and the QIAprep® Spin Miniprep Kit (Qiagen, Valencia, CA) for the isolation of plasmids. PCR was performed using the Phusion® PCR Master Mix (Finnzymes, Lafayette, CO) according to instructions provided by the manufacturer. All DNA modification enzymes used in this study were obtained from New England Biolabs (Ipswich, MA, USA) unless indicated differently.

### Culture media formulation

Bacto™ Tryptic Soy Broth without Dextrose (TSB, Becton Dickinson, Sparks, MD) was used as a rich culture medium for *R. eutropha* strains. For the growth of all *R. eutropha* strains, 10 μg/mL gentamycin sulfate (Gm10) was added. For the growth of *R. eutropha* strains containing a plasmid conferring a kanamycin resistance, 300 μg/mL kanamycin sulfate (Kan300, Calbiochem, USA) was added to the media. Minimal medium for *R. eutropha* was composed of 4.0 g/L NaH_2_HPO_4_, 0.45 g/L K_2_SO_4_, 0.39 g/L MgSO_4_, 62 mg/L CaCl_2_ and 1 mL per 1 L of a trace element solution (15 g/L FeSO_4_ ∙ 7H_2_O, 2.4 g/L MnSO_4_ ∙ H_2_O, 2.4 g/L ZnSO_4_ ∙ 7 H_2_O and 0.48 g/L CuSO_4_ ∙ 5 H_2_O dissolved in 0.1 M HCl). The initial pH of the minimal medium was 6.8. Nitrogen and carbon sources were added to minimal medium as indicated below. All *R. eutropha* strains and cultures were grown at a temperature of 30°C.

Lysogeny broth (LB) was used as culture medium for *E. coli* and in the mating and selection process of *R. eutropha* mutants. LB containing 50 μg/mL kanamycin (Kan50) was used for *E. coli* harboring plasmids. All *E. coli* cultures were grown at a temperature of 37°C.

### Creation of *R. eutropha* strains

For the creation of the plasmids pER1, pER2, pER3 and pER4 (see Table 2), *phaZ* genes were inserted into the plasmid pBBR1MCS-2, as discussed below. The genes *phaZ1phaZ2phaZ3* and *phaZ5* ([Handrick et al*.* 2000]; [York et al. 2003]) were amplified from *R. eutropha* H16 via PCR, using primers listed in Table 3. A double restriction digest was performed on both the plasmid pBBR1MCS-2 (see Table 2) and the inserts, using Kpn1 and Xba1 for the formation of pER1, pER2, pER4, or Sal1 and Xba1 for the formation of pER3. To construct a *phaZ3* deletion allele, two sets of primers were used to amplify two ~500 bp regions of DNA, directly flanking the *phaZ3* open reading frame. Primers phaZ3del1 and phaZ3del2 (Table 3) were used to amplify a fragment directly upstream of the *phaZ3* open reading frame. Primers phaZ3del3 and phaZ3del4 (Table 3) were used to amplify a fragment directly downstream of the *phaZ3* gene. The upstream insert was then digested with Sac1 and Pst1 and the downstream insert was digested with Pst1 and Xba1. The plasmid pGY46 (see Table 2) was digested with Sac1 and Xba1 to remove the *phaC* deletion allele. The purified digests of plasmid and desired inserts were ligated overnight at 15°C using T4 DNA ligase, following the manufacturer’s instructions. Conjugal transfer of plasmids into *R. eutropha* was performed by a standard procedure described previously ([Budde et al*.* 2010]; [Quandt and Hynes 1993]). For deletion of the *phaZ3* gene, *R. eutropha* strains H16 and Re1111 were used as parental strains. To determine the presence of the Δ*phaZ3* allele in the *R. eutropha* chromosome, diagnostic PCR was performed using the primers phaZ3delchkFW and phaZ3delchkRV (see Additional file 1: Figure S1). Strains positive for *phaZ3* deletion (Re2005 and Re2006, see Table 2) were used for further study.

**Table 3:** Primers used in this study

**Name/purpose**	Sequence(5’-3’)*
Deletion of *phaZ3* gene	
phaZ3del1	CAT A**GA GCTC**AT CGC TGC GGC AAC TTG GG
phaZ3del2	GAC A**CT GCAG**CA TGA AGA CTC CCG TAG GAA
phaZ3del3	GAT A**CT GCAG**AC TTA GCA TCG CCT GCC C
phaZ3del4	CAA G**TC TAGA**GA CGC GTG AAC AAG CTG
phaZ3delchkFW	CGG TGA ACC ATC GAA TTC
phaZ3delchkRV	CAA GGT TGA CCG CAG CAA G
Plasmid-based overexpression of *phaZ* genes	
PhaZ1 FW	GCA C**GG TAC C**AT GCT CTA CCA ATT GCA TGA G
Pha Z1 RV	GAT A**TC TAG A**TT ACC TGG TGG CCG AGG CCT G
Pha Z2 FW	CAT A**GG TAC C**AT GCT GTA CCA CGC CTA CCA
Pha Z2 RV	GCG T**TC TAG A**TT AGC TGC TGG TGT AGA TGG
Pha Z3 FW	CTA T**GT CGA C**AT GCT GTA CCA GCT CGT CGA G
Pha Z3 RW	CAA G**TC TAG A**CT AAG TAT CGT GCC CGG TCA
Pha Z5 FW	AAT T**GG TAC C**AT GGC GCT CTA TGC TCT GCG
Pha Z5 RW	GAA T**TC TAG A**TC AGT CGG CGC ACC GTT GAA
RT-PCR to quantify overexpression of *phaZ* genes	
PhaZ1 FW	CCATCAAGCTGCTCAAGGAT
Pha Z1 RV	CCAGTCGGTGACGTAGACCT
Pha Z2 FW	TCTACCTGGAAACCGTCAGC
Pha Z2 RV	CAGATATCGTCGCGTTCACC
Pha Z3 FW	GGACTATTGCCTGGATCTCG
Pha Z3 RV	AATTCCTGGAACACGAGCTG
Pha Z5 FW	ATGATCGAGGCAGGTTATCG
Pha Z5 RV	GACCTCGTCGATCTCAAAGC

*Restriction sites indicated in bold.

### RNA Extraction and reverse transcriptase quantitative PCR

For examination of *phaZ* gene overexpression in *R. eutropha* strains containing plasmid, total RNA extraction and reverse transcriptase quantitative PCR (RT-qPCR) were performed. *R. eutropha* strains containing plasmids were propagated in a 5 mL liquid culture of TSB/Kan300 + Gm10 at 30°C for approximately 40 h, and then used to inoculate a 50 mL culture of TSB/Kan300 + Gm10 to an OD_600_ of 0.1. The 50 mL cultures were incubated in a 500 mL shaking flask at 30°C and 200 rpm for ~8 h. The OD_600_ was measured and 2.5 OD units were transferred into a conical tube (BD Flacon). The cells were centrifuged at 5000 rpm and the supernatant was removed. The pellet was dried and quickly frozen by incubating in a slurry of dry ice and ethanol. Pellets were stored at −80°C until used for RNA extraction.

Total cellular RNA was extracted from *R. eutropha* strains using a method described previously ([Brigham et al. 2010]). Determination of RNA concentration and purity were performed on a Nanodrop 1000 (Thermo Scientific, Wilmington, DE) directly before using the purified RNA for RT-qPCR. RT-qPCR analysis was performed using the QuantiTect Reverse Transcription Kit (Qiagen, Valencia, CA) according to the manufacturer’s instructions. For each reaction, 100 ng of total cellular RNA was used. A reaction mixture without reverse transcriptase was used as a control for each sample. Product quantification was performed using the QuantiTect SYBR Green PCR Kit (Qiagen, Valencia, CA) according to the manufacturer’s instructions. The total reaction volume was 25 μL. One reaction mix without template cDNA was used as an additional control. For determination of cDNA concentration, the desired primer product was amplified via PCR using genomic DNA from *R. eutropha*. The PCR product was gel purified and serial dilutions were prepared (1:10, 1:10^2^, 1:10^3^, 1:10^4^, 1:10^5^, 1:10^6^, 1:10^7^, 1:10^8^). RT-qPCR was performed on selected aliquots of these DNA-dilutions in order to create standard curves (1:10^2^, 1:10^3^, 1:10^4^, and 1: 10^5^ for *phaZ1* and *phaZ2*; 1:10^2^, 1:10^3^, 1:10^4^, 1:10^5^, and 1:10^6^ for *phaZ3*; 1:10^4^, 1:10^5^, 1:10^6^, 1:10^7^, and 1:10^8^ for *phaZ5)*.

### Analysis of PHB production and depletion in *R. eutropha* strains harboring plasmid-borne *phaZ* genes

The strains H16/pBBR1MCS-2, H16/pER1, H16/pER2, H16/pER3, H16/pER4, Re2005/pBBR1MCS-2, Re2005/pER1, Re2005/pER2, Re2005/pER3 and Re2005/pER4 were initially propagated in 5 mL of TSB/Kan300 + Gm10 at 30°C for approximately 40 h.

For rich medium cultures, a 200 mL culture of TSB/Kan300 + Gm10 in a 1 L shaking flask was inoculated to an initial OD_600_ of 0.05. It was incubated at 30°C and 200 rpm for a total of 72 h. To determine the PHB content a 5 mL sample was taken immediately after inoculation (0 h). Following the initial sampling, culture aliquots of 30 mL were removed at intermittent time points for the same purpose.

For cultures in PHB production medium, cells from rich media precultures were washed once with a sterile 0.85% NaCl solution and used to inoculate a 200 mL culture of minimal medium enriched with 2% fructose and 0.05% NH_4_Cl to an initial OD_600_ of 0.05 in a 1 L culture flask. Cultures were incubated at 30°C with agitation at 200 rpm for a total of 72 h. Following inoculation, 5 mL of culture were removed to determine PHB content (0 h). After the first sampling, the following sample volumes were removed and used to determine PHB content of cells in culture: 25 mL after 4 h, 15 mL after 8 h and 10 h, 10 mL after 24, 48 and 72 h.

To formulate PHB consumption medium, 0.1% NH_4_Cl and no carbon source were added to *R. eutropha* minimal medium (see above). For PHB consumption cultures, after 72 h growth under conditions for PHB production, 100 mL aliquots of culture were removed, washed with a sterile 0.85% NaCl solution and transferred into 100 mL of PHB consumption medium in 500 mL culture flasks. These cultures were incubated at 30°C and 200 rpm for a total of 72 h. At intermittent time points in the culture, aliquots were removed to determine PHB content of the cells in culture.

The PHB content was assayed indirectly as crotonic acid by the high-pressure liquid chromatography (HPLC) method developed by Karr et al*.* ([Karr et al*.* 1983]) and performed in our laboratory as described previously ([Brigham et al. 2010]; [Budde et al. 2010]). Averages ($\overset{￣}{x}$) and standard deviation (σ) were calculated using the formulas (1) and (2). n is the number of experiments (n = 2),$x_{i}$ is PHB % of cell dry weight (%CDW) at a defined time point.

(1) $$\overset{￣}{\text{x}}=\frac{\sum x_{i}}{\text{n}}$$

(2) $$\text{σ}=\sqrt[{2}]{\frac{\sum {{(\text{x}}_{\text{i}}-\overset{￣}{\text{x}})}^{2}}{\text{n}-1}}$$

### Electron Microscopy

The following *R. eutropha* strains with deletions of one or more *phaZ* genes were imaged under a Jeol JEM-1200 EXII electron microscope (JEOL, Tokyo, Japan) at 59 kV: H16, Re1097, Re1107, Re1110, Re1112, Re2005, and Re2006 (see Table 2). Micrographs were acquired using an AMT V600 Camera (Advanced Microscopy Techniques, Danvers, MA, USA) and the corresponding software. NIS elements software (V 2.03, Nikon Instruments, Melville, NY) was used to analyze the images.

Growth of *R. eutropha* strains was carried out as follows. The strains were propagated in a 5 mL TSB/Gm10 liquid culture at 30°C for approximately 40 h. After 40 h, 1 mL of culture was used to inoculate a 50 mL liquid culture of TSB/Gm10 in a 250 mL shaking flask. This flask culture was incubated at 30°C with agitation of 200 rpm for 24 h. Cells from this culture were washed and used to inoculate a 25 mL minimal medium culture containing 2% fructose and 0.05% NH_4_Cl to an initial OD_600_ of 0.5. The culture was incubated in a 250 mL shaking flask at 30°C with agitation of 200 rpm. After approximately 22 h, 1.5 mL of cell culture were harvested from each flask and aliquots from each culture were taken and used for preparation of EM grids.

Embedding procedure of cells and preparation of EM grids was performed as follows. The cells harvested were washed twice with 50 mM HEPES buffer (pH 7) and enrobed in 2% molten Noble Agar. Once the agar had solidified, it was cut into 2 mm x 2 mm squares using a clean heated razor blade. The blocks were transferred into a microcentrifuge tube, resuspended in 0.5 mL 2% glutaraldehyde and stored for 1–2 h at room temperature. The blocks were washed three times in HEPES buffer (50 mM, pH7), resuspended in 0.5 mL 2% OsO_4_ and stored at 4°C for 1–2 h. Then, the blocks were washed three times in HEPES buffer, resuspended in 1% uranyl acetate and incubated for 1 h at room temperature. The blocks were dehydrated using 1 mL each of a graded ethanol series (25% vol., 50% vol., 75% vol., 100% vol.) incubating for 15 min each. The blocks were then resuspended in 1 mL of a 50% vol. LR White resin (London Resin Company, Berkshire, UK) in anhydrous ethanol and stored at 4°C until the blocks sink to the bottom of the tube. After that, the blocks were resuspended in 1 mL 100% LR White resin, placed into gelatin capsules containing LR White resin and incubated at 60°C over night to allow resin to polymerize. The capsules were shaped trimming away the exterior polymer with a razor blade, creating a trapezoid shape. 0.35 μm thick thin sections were cut at a speed of 1 mm/s using a Reichert Ultracut E microtome (Leica Microsystems, Bannockburn, IL, USA). Slices were collected on the carbon site of a copper grid.

Electron micrograph images were analyzed following the principles of stereology. A homologous sample, random slice selection and random orientation of cells on the slide were assumed. According to the Delesse principle, the area ratio of smaller elements randomly arranged in a bigger area to the big area equals the volume ratio of small elements to the bigger volume ([Delesse 1848]). This principle is applicable to microscopy and for our experiment setup it means:

(3) $$\frac{\sum \text{granulearea}}{\sum \text{cellarea}}=\frac{\sum \text{granulevolume}}{\sum \text{cellvolume}}$$

The mechanistic error determining the cell and granule area was estimated by 10fold determination of the cell and granule area of a randomly picked cell. Area ratio was determined for each of the measurements. The average of the granules per cell ($\overset{￣}{x}$) was calculated, as well as the standard deviation (σ) and the standard error ($\sigma_{\overset{￣}{x}}$) and the standard error in percent ($\sigma_{\overset{￣}{x}}$ (%)) according to formulas (4) through (7).

(4) $$\overset{￣}{x}=\frac{\sum n_{granules}}{n_{cells}}$$

(5) $$\text{σ}=\sqrt[{2}]{\frac{{{\sum (\text{x}}_{\text{i}}-\overset{￣}{\text{x}})}^{2}}{\text{n}-1}}$$

(6) $${\text{σ}}_{\overset{￣}{\text{x}}}=\frac{\text{σ}}{\sqrt[{2}]{\text{n}}}$$

(7) $${\text{σ}}_{\overset{￣}{\text{x}}}(\%)=\frac{{\text{σ}}_{\overset{￣}{\text{x}}}}{\overset{￣}{\text{x}}}100\%$$

For each strain the number of granules per full size cell picture (n_granules_) was determined as well as the total number of full length cells (n_cells_, or n in formulas 5 and 6). A cell with a length to width ratio of at least 3:1 was assumed to be a full grown cell. The arithmetic mean of granules per cell ($\overset{￣}{x}$) and the standard deviation (σ) and the standard error ($\sigma_{\overset{￣}{x}}$) were determined for each strain using the formulas 4, 5 and 6. Assuming a Gaussian normal distribution of the granule number per cell, the average of the whole population (μ) would be $\text{μ}=\overset{￣}{\text{x}}\pm 2\text{σ}$with a probability of 68% and $\text{μ}=\overset{￣}{\text{x}}\pm 2\text{σ}$ with a probability of 95% ([Rudolf and Kuhlisch 2008]). CS3 (Version 10.0, Adobe, San Jose, CA) and Paint.NET V3.5.5 (Washington State University/Microsoft, Pullman/Redmond, WA) were used to crop photographs used in this work.

## Results

### Plasmid construction and overexpression of *phaZ* genes

The plasmids pER1, pER2, pER3 and pER4 were constructed with the goal of overexpression of *R. eutropha* intracellular and putative intracellular *phaZ* genes in the wild type strain, H16, as well as complementation of PHB utilization deficiencies in the *phaZ* mutant strains. As discussed in Materials and Methods, the broad host-range vector pBBR1MCS-2 was used as a parental plasmid to construct the *phaZ* overexpression plasmids. Upstream of the multiple cloning site of pBBR1MCS-2 is a P_*lac*_ promoter that typically allows for expression of cloned genes in *E. coli* ([Kovach et al*.* 1995]). However, this promoter has been demonstrated to drive expression of genes in an *R. eutropha* host strain under growth and PHB production conditions ([Fukui et al*.* 2011]). In this study, the *phaZ* genes were cloned into the vector so they are expressed under the P_*lac*_ promoter. To demonstrate overexpression of *phaZ1, phaZ2, phaZ3* or *phaZ5* genes in strains of *R. eutropha* containing the prerequisite plasmid, mRNA levels were compared in strains with plasmid versus strains containing empty vector by RT-qPCR and consequent determination of cDNA concentration. Cells were therefore grown in rich medium cultures for approximately 8 h, and total cellular RNA was harvested from cells for detection of *phaZ* genes by RT-qPCR.

A significantly higher concentration of *phaZ*-specific cDNA was found in H16 strains containing plasmids pER1-4 than in the control (empty vector alone) strain H16/pBBR1MCS-2 (Table 4). These observations suggest that *phaZ* gene is overexpressed in the plasmid containing strains. In H16/pBBR1MCS-2, no cDNA from mRNA of the *phaZ2* or *phaZ3* genes could be detected under the given conditions. This is likely because of the low levels of expression of these two genes under rich medium growth conditions (Sinskey laboratory, unpublished data). Concentration of *phaZ5* cDNA from the RT-qPCR experiment was very low in H16/pBBR1MCS-2 (Table 4), likely also due to low gene expression under conditions tested. Expression of *phaZ* genes was also examined under PHB production and PHB consumption conditions. In both of these conditions, strains containing plasmids pER1, pER2, pER3, or pER4 demonstrated overexpression of their respective *phaZ* genes as compared to the control (Additional file 2: Figure S2).

**Table 4:** **Concentration of*phaZ1*-*phaZ5*cDNA [ng/μL] detected in RT-qPCR for H16/pBBR1MCS-2, H16/pER1, H16/pER2, H16/pER3, H16/pER4 amplified from 100 ng of RNA**

**Gene**^**a**^	**Plasmid present**^**b**^	**Concentration of cDNA [ng/μL]**^**c**^
*phaZ1*	pER1	4.21E-06
		3.05E-06
*phaZ1*	pBBR1MCS-2	3.07E-07
		2.63E-07
*phaZ2*	pER2	1.22E-04
		6.45E-05
*phaZ2*	pBBR1MCS-2	nd^d^
		nd^d^
*phaZ3*	pER3	3.68E-07
		1.01E-07
*phaZ3*	pBBR1MCS-2	nd^d^
		nd^d^
*phaZ5*	pER4	3.07E-07
		6.35E-07
*phaZ5*	pBBR1MCS-2	2.92E-09
		8.48E-10

^a^Indicates the primer pair of the corresponding *phaZ* gene used in the RT-qPCR reaction.

^b^Indicates the overexpression plasmid, or empty vector (pBBR1MCS-2), present in the *R. eutropha* H16 host strain.

^c^Top and bottom numbers of each sample represent results of two different experiments (ex. „4.21E-06“ corresponds to 4.21 × 10^-6^).

^d^Transcript not detected by RT-qPCR.

### Examination of PHB content of *R. eutropha* strains overexpressing *phaZ* genes and grown under different conditions

**Figure 1:**
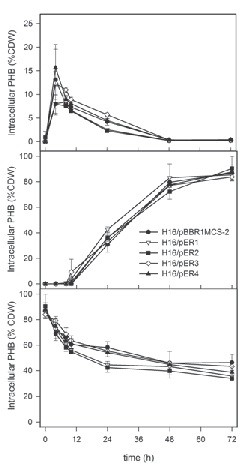
**PHB content as percentage of cell dry weight (CDW) in*R. eutropha*cultures grown in (A) TSB (rich) medium, (B) PHB production medium (minimal medium with 0.05 w/v% NH**_**4**_**Cl and 2 w/v% fructose), and (C) PHB consumption medium (minimal medium with 0.1 w/v% NH**_**4**_**Cl and no exogenous carbon source).** PHB content for the strains H16/pBBR1MCS-2 (vector alone control), H16/pER1 (overexpressing *phaZ1*), H16/pER2 (overexpressing *phaZ2*), H16/pER3 (overexpressing *phaZ3*), H16/pER4 (overexpressing *phaZ5*) are shown here. Values represented in these graphs are the averages of duplicate experiments. Error bars indicate standard deviation.

In rich medium (Figure 1A), the average intracellular PHB content reached its highest level (between 8% and 18% of cdw) after 4 h of cultivation for all strains. After this climax, the PHB content declined slightly over the next 5 h. After 24 h, the intracellular PHB content had decreased to 2%-6% cdw. After 48 h, the PHB was completely depleted. No significant differences could be detected between the different plasmid-bearing strains and the control. This transient PHB accumulation in rich media cultures was similar to observations from previous works ([Tian et al. 2005a]; [York et al. 2003]).

In growth medium formulated to promote PHB production (Figure 1B), no significant PHB production could be observed during the first 10 h, likely corresponding to cell growth and the presence of extracellular nitrogen. After this, the PHB content steadily increased to a concentration of 70%-80% cdw after 48 hours. The final PHB content (after 72 h) for all strains was ~80-90 % cdw. No significant differences could be detected between the different plasmid bearing strains and the control, indicating that overexpression of *phaZ* genes has no effect on intracellular PHB accumulation in *R. eutropha*.

In PHB consumption medium (Figure 1C), minimal medium with no exogenous carbon, the intracellular PHB accumulated during the PHB production phase was used as a carbon source. A rapid decrease in PHB content from 90% cdw to ~60% cdw could be observed during the first 10 h. From 10 h to 72 h, the PHB content decreased more moderately from 60% to 40%-50% cdw. After 24 h, the PHB content in the strains H16/pER1 and H16/pER2 had already decreased to 40% cdw, whereas ~60% cdw intracellular PHB still remained in the other strains. This observation suggests that the overexpression of *phaZ1* and *phaZ2* resulted in an increased rate of intracellular PHB utilization over the first 24 h. After 24 h, no significant differences could be detected between the different plasmid-bearing strains and the control.

### Complementation of *R. eutropha* strain Re2005

**Figure 2:**
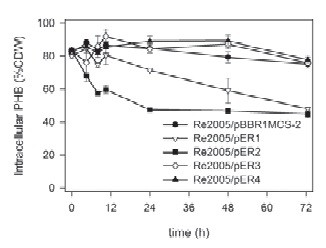
**PHB content of Re2005 (Δ*phaZ1*Δ*phaZ2*Δ*phaZ3*Δ*phaZ5*) strains, complemented with different*phaZ*genes*in trans*, incubated in PHB consumption medium (minimal medium with 0.1 w/v% NH**_**4**_**Cl and no exogenous carbon source).** PHB contents for the strains Re2005/pBBR1MCS-2, Re2005/pER1 (+*phaZ1*), Re2005/pER2 (+*phaZ2*), Re2005/pER3 (+*phaZ3*), Re2005/pER4 (+*phaZ5*) over the course of a 72 h incubation time are shown here. Values represented in this graph are the average of triplicate experiments. Error bars indicate the standard deviation.

We also examined PHB content in Re2005 (Δ*phaZ1*Δ*phaZ2*Δ*phaZ3*Δ*phaZ5*) cells containing plasmids pER1-4 under PHB utilization conditions (Figure 2), attempting to complement the PHB utilization phenotype of the strain. Intracellular PHB accumulated under polymer production conditions remained approximately the same (~80% cdw) in Re2005/pER3, Re2005/pER4 and Re2005/pBBR1MCS2. This suggests that addition of *phaZ3* or *phaZ5* genes to a Re2005 host *in trans* does not result in complementation of the PHB utilization phenotype. In PHB utilization conditions, for Re2005/pER1 the intracellular PHB content did not decrease for the first 10 h, and then exhibited a linear decrease from ~80% of cdw to ~50% of cdw over the next 62 h. This observation suggests that *phaZ1* will complement the phenotype of Re2005 when introduced *in trans.* In Re2005/pER2 a steep decline from ~80% cdw to ~55% cdw was observed during the first 8 h. From 10 h to 24 h, the PHB content decreased more moderately from ~55% cdw to ~45% cdw. After that, it remains stable at ~45% cdw. The final concentration for Re2005/pER2 is reached after ~24 h, whereas Re2005/pER1 commences PHB consumption between 10 h-24 h, so Re2005/pER2 seems to have reached almost the final PHB level when Re2005/pER1 has just begun PHB consumption. These observations could imply that PhaZ2, expressed from pER2, is more efficient in mobilizing PHB from granules than is PhaZ1, expressed from pER1. In summary, these observations suggest that the overexpression of either *phaZ1 or phaZ2* in this strain restore the ability of Re2005 to utilize PHB under the given conditions, whereas the overexpression of *phaZ3* and *phaZ5* appears to have no effect.

### Electron Microscopy

**Figure 3:**
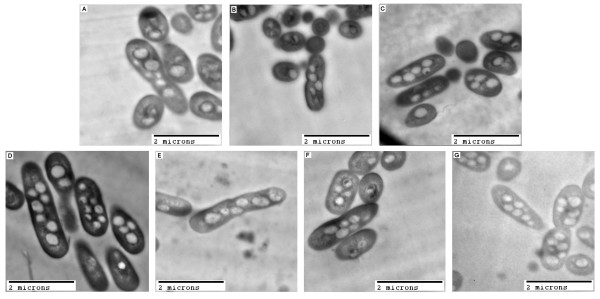
**Electron micrographs of*R.eutropha*strains after ~22 h of growth in PHB production medium:** (**A**) H16 (wt), (**B**) Re1097 (Δ*phaZ1*), (**C**) Re1107 (Δ*phaZ5*), (**D**) Re1110 (Δ*phaZ2*), (**E**) Re1112 (Δ*phaZ1*Δ*phaZ2*), (**F**) Re2005 (*ΔphaZ1ΔphaZ2ΔphaZ3ΔphaZ5*), (**G**) Re2006 (*ΔphaZ3*). Bar = 2 μm.

Electron microscopy was performed to determine the effect of *phaZ* deletions on granule count, size, and shape in *R. eutropha*. It is already an established method to visualize *R. eutropha* H16 cells and granules under different growth and PHB production conditions ([Tian et al. 2005a,2005b]). The amount (Tables 5 and 6) and quality (examples in Figure 3) of *R. eutropha* cell and granule images in the obtained micrographs were suitable for statistical analysis.

**Table 5:** **Area ratios, standard error in percent (**$\sigma_{\overset{￣}{x}}$**(%)) and sample sizes (n) of the area ratios for H16, Re1097 (Δ*phaZ1*), Re1107 (Δ*phaZ5*), Re1110 (Δ*phaZ2*), Re 1112 (Δ*phaZ1*Δ*phaZ2*), Re2005 (Δ*phaZ1*Δ*phaZ2*Δ*phaZ3*Δ*phaZ5*), Re2006 (Δ*phaZ3*)**

	**Area ratio**	**standard error in percent, (**$\sigma_{\overset{￣}{x}}$**, (%))**	**Sample size (n)**
H16	0.25	2.01	97
Re1097	0.28	2.01	106
Re1107	0.29	2.01	98
Re1110	0.30	2.01	101
Re1112	0.32	2.01	100
Re2005	0.22	2.01	99
Re2006	0.27	2.01	101

**Table 6:** **Average granule count per cell (**$\overset{￣}{x}$**), standard error (**$\sigma_{\overset{￣}{x}}$**) and sample size (n) of the area ratios for H16, Re1097 (Δ*phaZ1*), Re1107 (Δ*phaZ5*), Re1110 (Δ*phaZ2*), Re 1112 (Δp*haZ1*Δ*phaZ2*), Re2005 (Δ*phaZ1*Δ*phaZ2*Δ*phaZ3*Δ*phaZ5*), Re2006 (Δ*phaZ3*)**

***R. eutropha***** strain**	**Average (**$\overset{￣}{x}$**) granules/cell**	**Standard error (**$\sigma_{\overset{￣}{x}}$**)**	**Sample size (n)**
H16	5.56	0.54	18
Re1097	5.41	0.63	18
Re1107	5.55	0.47	20
Re1110	6.83	0.40	24
Re1112	7.06	0.45	17
Re2005	5.39	0.37	18
Re2006	7.00	0.46	16

In Table 5, the area ratios of electron micrograph images of each strain are listed. The Delesse principle tells us that the area ratio of smaller elements (in this case, 2-dimentional representations of granules) randomly arranged in a larger space (in this case, the 2-dimensional representation of the cell) compared to the area of that larger space equals the volume ratio of the small elements (granules) to the bigger area (cell). In short, we can estimate the amount of space inside the cell of each strain taken up by PHB granules, and estimate if the deletion of *phaZ* genes plays a role in altering that ratio. The mechanistic standard error, in percent (${\text{σ}}_{\overset{￣}{\text{x}}}$ (%)), for the determination of granule/cell area ratio was found to be 2.01% (Table 5). The data shown in Table 5 indicate that strains Re1110 (Δ*phaZ2*) and Re1112 (Δ*phaZ1*Δ*phaZ2*) exhibit a slight increase in the granule/cell area ratio. This suggests that the loss of PhaZ2 has a slight effect on the granule/cell area ratio, indicating a potential function of PhaZ2 in shaping and remodeling PHB granules in the *R. eutropha* cell. The quadruple deletion strain, Re2005 (Δ*phaZ1*Δ*phaZ2*Δ*phaZ3*Δ*phaZ5*) exhibits a slight decrease in the area ratio, suggesting other PhaZ enzymes may also have granule shaping and remodeling functions.

While it has been shown that the presence or absence of PHB depolymerase enzymes does not control the overall amount of PHB in the cell, a role of these enzymes in granule formation is strongly suggested, both by the presence of PhaZ proteins on the surface of PHB granules ([Uchino and Saito 2006]; [Uchino et al. 2007]) and the alteration of granule/cell area ratios in *phaZ* mutant strains. We sought to determine whether PHB depolymerase genes played a role in the number of granules present inside the cell. Table 6 shows the average granule count of *R. eutropha* wild type and *phaZ* mutant strains. Individual standard errors for the average granule count ranged between ~6% and ~12%. Quantitative data could be derived from the statistical analysis: In Re1110 (Δ*phaZ2*) and Re1112 (Δ*phaZ1*Δ*phaZ2*), more granules were observed per cell than in the wild type strain, H16. These observations suggest that PhaZ enzymes play roles in granule formation, potentially via control of the number of granules per cell. Strain Re2006 (Δ*phaZ3*) showed a high granule number per cell, whereas the ratio of granule to cell area was only slightly higher than in the wild type. This suggests that the presence of PhaZ3 in the *R. eutropha* cell may affect granule formation through control of intracellular granule count. Granule count in Re1097 and Re1107 did not significantly differ from the wild type, whereas the cell/granule area ratio was slightly higher. In general, these observations suggest that the absence of specific *phaZ* genes affects granule number and architecture in different strains.

## Discussion

Data shown here indicate that overexpression of *phaZ1* and *phaZ2* genes has an effect on PHB consumption in *R. eutropha* strains H16 and Re2005 (Figures 1C and 2). The active role of these enzymes in PHB consumption was shown earlier by analysis of PHB content in *phaZ1* and *phaZ2* deletion strains ( [York et al. 2003]). In *R. eutropha* H16, overexpression of these genes seems to have a minor effect on PHB homeostasis. Utilization of PHB appears to be accelerated in H16/pER1 and H16/pER2 (Figure 1C), but the overexpression of *phaZ1* and *phaZ2* does not seem to have a significant effect on the final PHB content after 72 h in PHB utilization medium. In Re2005/pER2, PHB levels similar to those of the wild type strain are almost reached when the strain is incubated in PHB consumption medium. Furthermore, the final PHB content is almost reached in Re2005/pER2 (*i.e.* the cells have mobilized near maximum amount of PHB under utilization conditions) when Re2005/pER1 begins polymer consumption. This observation suggests that *phaZ2* expression can complement the phenotype of the quadruple depolymerase mutant, in terms of overall PHB mobilization. This might also indicate that PhaZ1 acts upon products of PhaZ2 under conditions examined in this work. The opposite – PhaZ2 acting upon products of PhaZ1 activity - had been suggested in an earlier work, based upon analysis of gene deletion mutants ([York et al. 2003]). Furthermore, in Re2005, expression of *phaZ1 in trans* resulted in linear kinetics of PHB consumption over time, whereas expression *in trans* of *phaZ2* seemed to bring about exponential kinetics of utilization. This suggests different kinetics of PHB depolymerase activity of these two enzymes. It is possible that the manner of cooperation between PhaZ1 and PhaZ2 enzymes is dependent on the depolymerase enzyme associated with the PHB granule.

According to York, et al*.* ([York et al. 2003]), a single deletion of the *phaZ2* gene does not have an effect on the total amount of PHB mobilized in *R. eutropha* cultures. This observation, along with data shown here, suggests that PhaZ2 enables PHB consumption but that it is not necessarily required in presence of PhaZ1. Microarray data ([Brigham et al. 2012]) suggest that expression of *phaZ1* is slightly upregulated during PHB production in comparison to growth and slightly down regulated during consumption in comparison to PHB production. Expression of *phaZ2* appears to be high under PHB production conditions. It remains unclear why individual overexpression of *phaZ1* and especially *phaZ2* in *R. eutropha* H16 does not appear to result in a more pronounced effect on intracellular PHB content under polymer production conditions. One possible explanation is that action of PhaZ2 causes a rearrangement of PHB polymer chains and then contributes degradation in an early stage of PHB consumption. Observed differences in granule/cell area ratios (Table 5) and granule counts (Table 6) in strains Re1110 (Δ*phaZ2*) and Re1112 (Δ*phaZ1*Δ*phaZ2*), when compared to wild type, suggest that PhaZ2 could indeed have a granule formation/remodeling role in the *R. eutropha* cell.

From the calculated area ratios, we can conclude that Re1110 (*ΔphaZ2*) and Re1112 (*ΔphaZ1 ΔphaZ2*) have a higher granule volume per cell volume compared to the wild type. This increased volume ratio can be attributed to higher granule counts (per cell) in these strains in comparison to wild type. Analysis of the PHB content in these strains ( [York et al. 2003]) has shown that Re1110 and Re1112 contain the same amounts of PHB as the wild type strain, after 24 h growth in PHB production medium. Accordingly, the same amount of PHB would be distributed over a larger number of granules. Preliminary fluorescence microscopy data suggests that strain H16/pER2 (with expression of *phaZ2 in trans*) displayed a lower granule count than the wild type and the other overexpressing strains (data not shown). Table 6 shows that a deletion of *phaZ2* results in a slight increase of granule number per cell. These two observations suggest an effect of PhaZ2 on the 3-dimensional architecture of the granules or, more specifically, on their density.

While it is clear from our data and previously published data ([York et al. 2003]) that PhaZ1 and PhaZ2 play major roles in PHB homeostasis in *R. eutropha,* data from the present work suggest roles for the two previously uncharacterized (or little characterized) depolymerases, PhaZ3 and PhaZ5. Neither PhaZ3 nor PhaZ5 were shown to play a noticeable role in PHB mobilization, suggesting their roles in PHB homeostasis are more subtle. According to the calculated area ratios, a slight but significant decrease in granule volume per cell volume is estimated in Re2005 (*ΔphaZ1 ΔphaZ2 ΔphaZ3 ΔphaZ5*) compared to wild type (Table 5). Moreover, a low granule count was observed in this strain in comparison to wild type (Table 6). Re2006 (Δ*phaZ3*) displayed a high granule count, but the granule volume per cell volume was estimated to be slightly but significantly higher than that of the wild type. Analysis of the PHB content in these strains (data not shown) suggests that Re2005 and Re2006 have the same PHB content as wild type under the conditions tested. This indicates higher granule density in Re2005 and Re2006. It also supports the hypothesis that PhaZ enzymes, specifically the newly characterized PhaZ3, are involved in 3D architecture of granules. Re1107 (Δ*phaZ5*) exhibited a higher area ratio than wild type, suggesting increased granule volume per cell volume, even though granule count was similar between the two strains. According to previous results ([York et al. 2003]), the intracellular PHB contents between the two strains are similar. This suggests that Re1107 has a lower granule density than wild type, suggesting a role for PhaZ5 in PHB granule architecture remodeling. Thus, while PhaZ3 and PhaZ5 enzymes do not appear to participate in PHB mobilization in a significant way, they do appear to affect the granule architecture inside the cell. It is also possible that crystallinity of intracellular PHB may be altered in these mutant strains.

Over the past three decades of research, many factors have been linked with PHB granule formation in *R. eutropha*. Initially, the PHB synthase enzyme was demonstrated to be associated with granules ([Gerngross et al*.* 1993]), and obviously plays a major role in formation of the intracellular bodies. Phasin proteins were also found to be associated with granules, with their primary and secondary structures making them well-suited for separating the hydrophobic polymer from the hydrophilic cytoplasmic contents ([Pötter et al*.* 2002]; [York et al. 2001]). PhaR protein, also thought to be granule-associated ([Yamada et al. 2007]; [Yamashita et al. 2006]), regulates the expression of the most abundant phasin protein, PhaP1, and has been shown to be able to bind DNA at a specific locus ([York et al. 2002]), as well as non-specifically on hydrophobic PHB ([Yamada et al. 2007]). Recently, a new type of protein factor, PhaM was discovered in *R. eutropha* ([Pfeiffer et al*.* 2011]). The role of PhaM was localization of granule initiation and provided a link between granule formation and the nucleoid of the cell, suggesting that PHB granules form not at the site of the cytoplasmic membrane, but in the cytoplasm itself ([Pfeiffer et al. 2011]). It was also shown that the intracellular depolymerase PhaZ1 was associated with granules, and functioned in thiolysis of 3HB-CoA from PHB chains in the granules ( [Uchino and Saito 2006]; [Uchino et al. 2007]). PhaZ2 was also shown to play a role in PHB degradation ([York et al. 2003]), suggesting its presence on the surface of the PHB granule. From our studies discussed here, it is tempting to speculate a PHA granule-associated role for the other known intracellular PHB depolymerases, PhaZ3 and PhaZ5, perhaps assisting with remodeling of the polymer chains within the granule. Given the complexity of the protein machinery surrounding the PHB granule, allowing for polymer to be synthesized or broken down as extracellular nutrient availability dictates, it can be said that the polymer granules truly are bacterial intracellular organelles ( [Jendrossek 2009]).

Uncovering the biology of PHB homeostasis in the model organism, *R. eutropha*, is advantageous for future studies on applications of the polymer. It will allow for development of bioprocesses with recovery of a maximum amount of useful biodegradable polymer from cells with minimal effort, allowing for easier polymer characterization and biocompatibility studies. Given the attractiveness of PHA in medical and tissue engineering applications, a thorough understanding of *R. eutropha* PHA biosynthesis could lead to a new role for the bacterium as an attractive industrial organism.

## Competing interests

The authors declare that they have no competing interests.

## Supplementary Material

Additional file 1 **Figure S1.** Confirmation of deletion of the *phaZ3* gene from the *R. eutropha* chromosome. Click here for file

Additional file 2 **Figure S2.** Quantitative ratios of *phaZ* transcript amounts in H16 strains containing pBBR1MCS-2 (vector only), pER1 (with *phaZ1* gene), pER2 (with *phaZ2* gene), pER3 (with *phaZ3* gene), or pER4 (with *phaZ5* gene). Click here for file
