# Supplementary material for: Examination of PHB Depolymerases in Ralstonia eutropha: Further Elucidation of the Roles of Enzymes in PHB Homeostasis
Source: AMB Express. 2012 Apr 26;2:26. doi: 10.1186/2191-0855-2-26 (PMC3430594; doi:10.1186/2191-0855-2-26)
Supplement: Additional file 1 — Figure S1. Confirmation of deletion of the phaZ3 gene from the R. eutropha chromosome. [file 2191-0855-2-26-S1.doc]

**Additional File 1.**

**
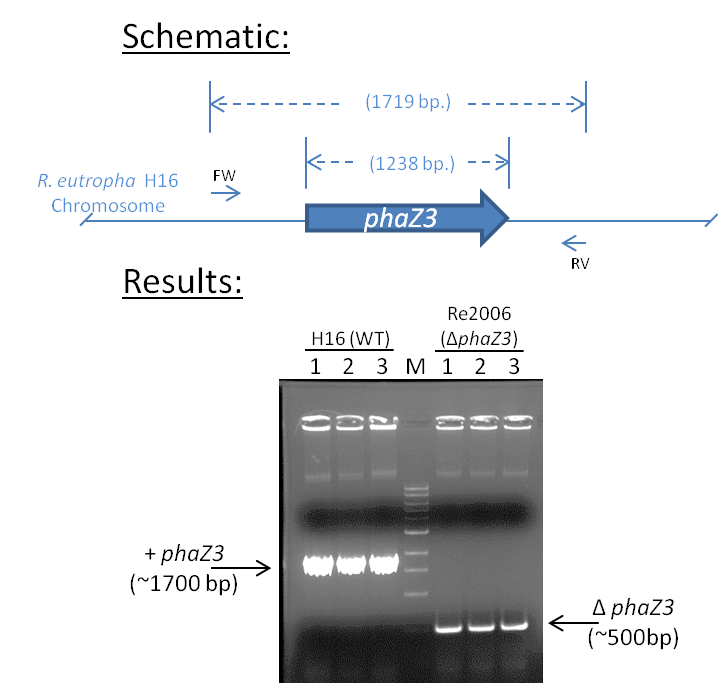
**

Confirmation of deletion of the *phaZ3* gene from the *R. eutropha* chromosome. Shown (top) is a schematic of a PCR experiment using phaZ3delchkFW (denoted as FW) and phaZdelchkRV (denoted as RV) primers that flank the *phaZ3* gene in the *R. eutropha* chromosome. If the *phaZ3* gene is present on the chromosome, then a ~1700bp PCR product will be seen on an agarose gel of the PCR product (bottom, left). However, if *phaZ3* has been successfully and completely deleted, then a ~500bp PCR product will be seen on an agarose gel (bottom, right). The DNA size marker used in this experiment is the 1kb DNA Ladder from New England Biolabs (catalog# N3232S; Ipswich, MA, USA). See Table 3 for sequence of primers used here.
