# Supplementary material for: Examination of PHB Depolymerases in Ralstonia eutropha: Further Elucidation of the Roles of Enzymes in PHB Homeostasis
Source: AMB Express. 2012 Apr 26;2:26. doi: 10.1186/2191-0855-2-26 (PMC3430594; doi:10.1186/2191-0855-2-26)
Supplement: Additional file 2 — Figure S2. Quantitative ratios of phaZ transcript amounts in H16 strains containing pBBR1MCS-2 (vector only), pER1 (with phaZ1 gene), pER2 (with phaZ2 gene), pER3 (with phaZ3 gene), or pER4 (with phaZ5 gene). [file 2191-0855-2-26-S2.doc]

**Additional File 2.**

**
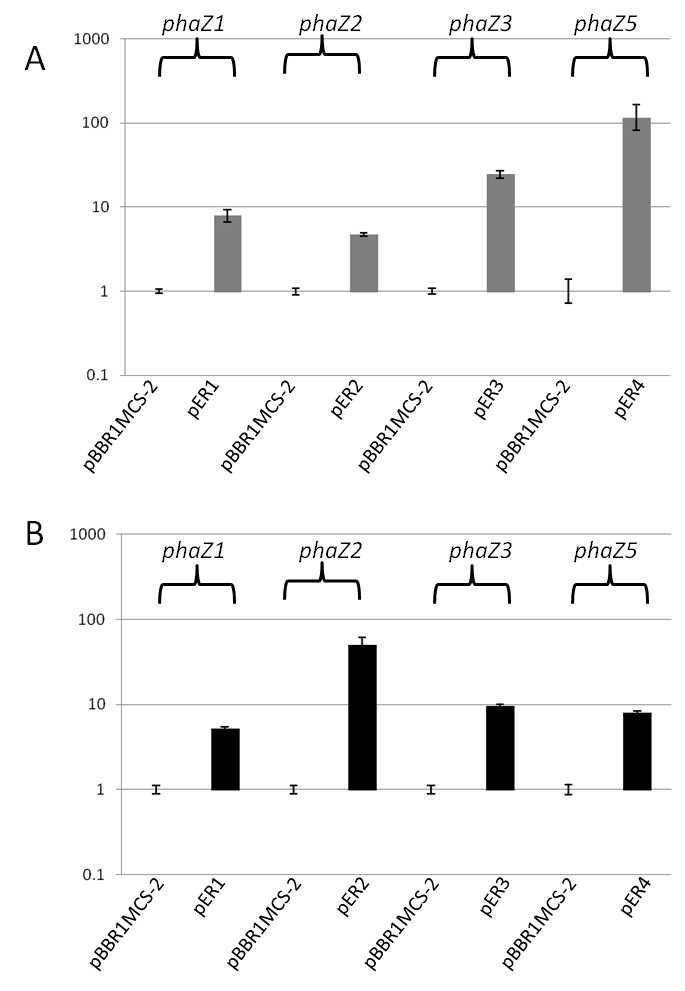
**

Quantitative ratios of *phaZ* transcript amounts in H16 strains containing pBBR1MCS-2 (vector only), pER1 (with *phaZ1* gene), pER2 (with *phaZ2* gene), pER3 (with *phaZ3* gene), or pER4 (with *phaZ5* gene). Results are grouped according to which transcript was detected in each sample (brackets). A ratio of 1 indicates that transcript levels are similar to the vector only control, and a ratio of >1 indicates increased expression of the *phaZ* gene beyond wild type levels. Transcript ratios were determined under PHB producing conditions (A) and PHB consumption conditions (B). The *Y*-axis in both (A) and (B) is the ratio [*phaZ* mRNA in H16/pER1, 2, 3, or 4]/[*phaZ* mRNA in H16/pBBR1MCS-2].

**Additional File 2, Materials and Methods.** For examination of *phaZ* gene expression or overexpression in *R. eutropha* H16 strains containing plasmids, total RNA extraction and reverse transcriptase PCR (RT-PCR) were performed. *R. eutropha* strains containing plasmids were propagated in a 5 mL liquid culture of TSB/Kan300+Gm10 at 30°C for 18 h, and then used to inoculate a 50 mL culture of minimal medium supplemented with Kan300+Gm10 to an OD_600_ of 0.1. The 50 mL cultures were incubated in a 500 mL shaking flask at 30°C and 200 rpm for ~18 h. The OD_600_ was measured and 2.5 OD units were transferred into a microcentrifuge tube (Eppendorf). The cells were treated with RNAprotect reagent (QIAgen, Valencia, CA) and then centrifuged at 13,000 rpm and the supernatant was removed. Cell pellets were then used to isolate RNA for RT-PCR experiments. Cultures were incubated in a 500 mL shaking flask at 30°C and 200 rpm for an additional 54 h, and then harvested. Cells were washed twice with 0.85% NaCl and resuspended in PHB utilization medium (higher nitrogen content, no carbon, see Materials and Methods) supplemented with Kan300+Gm10 and incubated at 30°C and 200 rpm. After ~18 h of incubation, cells were harvested as discussed above.

Total cellular RNA was extracted from *R. eutropha* strains using a method described previously (Brigh*am et a*l., 2010) with slight modifications. Determination of RNA purity was performed by PCR using Phusion DNA polymerase (Finnzymes) and subsequent examination of DNA products on an agarose gel. If contaminating DNA was present in the RNA preparation, then DNase treatment (Roche) was performed, and RNA purity was again determined. Determination of RNA quantity was performed on a Nanodrop 1000 (Thermo Scientific, Wilmington, DE) directly before using the purified RNA for RT-PCR. RT-PCR analysis was performed using the QuantiTect SYBR Green RT-PCR Kit (Qiagen, Valencia, CA) according to the manufacturer’s instructions (with slight modifications to reagent amounts to accommodate 30 μL reactions). For each reaction, 200 ng of total cellular RNA was used. The total reaction volume was 30 µL. Ratios of *phaZ* transcript quantity from strains containing pER1, pER2, pER3, or pER4 were compared to transcript quantities from strains containing pBBR1MCS-2. As a control, samples were treated with RNase and then subjected to RT-PCR in order to see whether the expression levels due to any DNA contamination were negligible.

**Reference**

Brigham, C. J., C. F. Budde, J. W. Holder, Q. Zeng, A. E. Mahan, C. Rha & A. J. Sinskey, (2010) Elucidation of beta-oxidation pathways in Ralstonia eutropha H16 by examination of global gene expression. *J Bacteriol* **192**: 5454-5464.
